# Iron overload triggering ECM-mediated Hippo/YAP pathway in follicle development: a hypothetical model endowed with therapeutic implications

**DOI:** 10.3389/fendo.2023.1174817

**Published:** 2023-05-08

**Authors:** Lingjin Xia, Yupei Shen, Suying Liu, Jing Du

**Affiliations:** ^1^ National Health Commission of the People's Republic of China (NHC) Key Lab of Reproduction Regulation (Shanghai Institute for Biomedical and Pharmaceutical Technologies), School of Pharmacy, Fudan University, Shanghai, China; ^2^ Reproductive Medicine Center, Zhongshan Hospital, Fudan University, Shanghai, China

**Keywords:** follicle development, iron overload, ferroptosis, extracellular matrix (ECM), Hippo/YAP pathway

## Abstract

Disruption of iron homeostasis plays a negative role in follicle development. The dynamic changes in follicle growth are dependent on Hippo/YAP signaling and mechanical forces. However, little is known about the liaison between iron overload and the Hippo/YAP signalling pathway in term of folliculogenesis. Here, based on the available evidence, we established a hypothesized model linking excessive iron, extracellular matrix (ECM), transforming growth factor-β (TGF-β) and Hippo/Yes-associated protein (YAP) signal regarding follicle development. Hypothetically, the TGF-β signal and iron overload may play a synergistic role in ECM production via YAP. We speculate that the dynamic homeostasis of follicular iron interacts with YAP, increasing the risk of ovarian reserve loss and may enhance the sensitivity of follicles to accumulated iron. Hence, therapeutic interventions targeting iron metabolism disorders, and Hippo/YAP signal may alter the consequences of the impaired developmental process based on our hypothesis, which provides potential targets and inspiration for further drug discovery and development applied to clinical treatment.

## Introduction

The regulation of iron homeostasis is particularly indispensable for maintaining dynamic reciprocity of cellular biological redox (1). Disrupted iron metabolism triggers iron overload, which acts as a catalyst for redox reactions, leading to the accumulation of lipid peroxides that induce cytotoxicity (2, 3). Follicle development expresses rapid cell proliferation and increased steroid generation, which is essential for iron demands (4). Conversely, iron overload results in a loss of antioxidant defenses, which affects follicle development (5).

Primordial follicle pools are limited and mostly in a dormant state (6, 7). The development process cannot be reversed once primordial follicles are properly activated; this plays a decisive role in maintaining female reproductive life span (8–11). Follicle development goes through different stages, which involves a series of complex processes. Extracellular matrix (ECM), an essential component of the ovarian microenvironment (12) governed by the TGF-β pathway (13) and iron metabolism (14), can dynamically regulate follicle assembly, activation, and dormancy (15, 16). Iron overload in the follicular fluid of patients with endometriosis leads to impaired oocyte maturation, possibly because granulosa cells with ferroptosis releasing exosomes that inhibit oocyte development (17). Mice with ovarian endometriosis showed significant fibrosis in the ovary, accompanied by increased iron accumulation, resulting in follicular oxidative stress and a dramatically decrease in litter size (18). As an upstream effector, ECM controls the dynamics of the Hippo pathway (19), this is derived from the appreciation of mechanical operations that interfere with ovarian Hippo signal transduction and promote follicle growth (20). In reproductive diseases affecting follicle development such as premature ovarian insufficiency (POI), Yes-associated protein (YAP, also known as YAP1), a key effector of the Hippo pathway, has been reported as a susceptibility gene of it (21, 22). The interactive mode between the Hippo pathway and the TGF-β signal is crucial for follicle development events (20, 23, 24). Therefore, it is highly likely that crosstalk connecting iron metabolism and the two pathways regulates follicle fate. However, a body of evidence merely concentrates on how iron overload induces intracellular oxidative stress. This review explores novel potential therapeutic targets with clinical significance and application value in improving female fertility by deducing a hypothetical mechanism linking excessive iron with the ECM-mediated Hippo pathway in terms of follicle maturation and development.

## Method

We used multiple strategies to identify major research publications written in English before July 2022 regarding iron metabolism, extracellular matrix, Hippo/YAP pathway, and follicle development. Under the premise of ensuring the preciseness of this study, we have conducted extensive screening on PubMed and/or Google Scholar using the following keywords alone or in combination: iron, iron overload, ferroptosis, follicles, extracellular matrix (ECM), actin, collagen, fibronectin, mechanical transduction, mechanical stress, Hippo pathway, Transforming growth factor-β (TGF-β), SMAD, BMP, Hepcidin, Fibrosis, premature ovarian insufficiency (POI), drug therapy, coenzyme Q10, resveratrol, melatonin, etc. We also searched the bibliography for other related articles. Overall, we reviewed most of the relevant articles and included them appropriately. Figures were generated via www.biorender.com

## Mechanism of iron overload and ferroptosis

Ferroptosis is mediated by intracellular iron overload (25). As a new type of iron-dependent cell death, it is characterized by mitochondrial collapse, the compression of membrane contents, and the accumulation of lipid peroxidation (26, 27). Exposure to excess iron, or polyunsaturated fatty acids (PUFAs) such as arachidonic acid and adrenal acid that activate membrane remodeling enzymes such as acyl-CoA synthase long chain family 4 (ACSL4), which is involved in catalyzing the acetylation of long-chain PUFAs to produce lipid peroxides esterified by interaction with membrane phospholipids (27–29). Ferroptosis, therefore, is strongly driven by ACSL4 (30). Transferrin receptor (TFRC) is a type II transmembrane receptor responsible for the uptake of cellular iron through receptor-mediated endocytosis (31). Dysfunction of iron uptake, transport, and storage, a major contributor to ferroptosis sensitivity, results from over-active TFRC mediating the internalization of iron load (32–34). TFRC knockdown by the lentivirus system inhibits erastin-induced ferroptosis (26). Similarly, suppressed TFRC reduces the risk of ferroptosis induced by amino acid/cysteine (35). In addition, nicotinamide adenine dinucleotide phosphate (NADPH) oxidase (NOXs), including NOX1, NOX2, NOX3, and NOX4, produces oxidative radicals and is the main source of the intracellular lipid reactive oxygen species (ROS) (36). Increased ROS levels lead to the accumulation of lipid peroxidation and ferroptosis (37). The antioxidant glutathione (GSH) is a powerful scavenger of lipid peroxidation products and acts as a cofactor of glutathione peroxidase 4 (GPX4) to restrain the progress of ferroptosis (37). One of the main mechanisms of ferroptosis may be triggered by impaired GSH metabolism (38). High concentrations of homocysteine were reported to induce GPX4 methylation leading to oxidative stress and ferroptosis by promoting methylase expression (39).

## Negative role of iron overload in follicle development

Numerous research focus on the correlation between iron overload and follicle development confirmed in a variety of experimental models. Iron accumulation and ferroptosis may occur in the early stage of follicle growth (40). Direct evidence comes from Hu et al. Via establishing an iron overload model induced by ferric ammonium citrate (FAC), the impaired quality of porcine oocytes was observed (5). Li et al. (41) found that transferrin was significantly reduced with increased iron concentration (P <0.05) in follicle fluid samples of advanced endometriosis. Direct negative effects on mouse oocytes with *in vitro* maturation were determined (41). Decreased oocyte quality, impaired ovarian reserve, and reduced ability of embryonic development can be induced by excessive intracellular ROS accumulation (42–46). In women with thalassemia, iron overload has a toxic effect on the anterior pituitary, leading to hypogonadotropic hypogonadism, low gonadotropin secretion, reduced antral follicle count, and fertility ability (47, 48).

## Mechanism of iron overload occurrence in follicle development-related diseases

Abnormal follicle development is the main phenotype of the onset of POI and PCOS. Women with POI lose ovarian activity and develop endocrine disturbances before the age of 40 (49). Iatrogenic factors such as chemotherapy have toxic effects on the reproductive system of young women (50). The main pathogenic mechanism may be through inducing oocyte apoptosis or destroying granulosa cell function, leading to primordial follicle loss, increase in atretic follicle and ovarian tissue fibrosis (51, 52). Accumulation of iron and lipid peroxide are involved in the pathological progress of fibrosis diseases (53, 54). Du et al. reported the association between ferroptosis and POI; this is mediated by cisplatin (55). The process involves lipid peroxidation promoting ferroptosis in granulosa cells, thus causing follicle development disorders and ovarian tissue fibrosis, ultimately perturbing ovarian function and fertility (55). Wang et al. (22)reported that BNC1 gene deficiency triggers oocyte ferroptosis via the NF2-YAP pathway and the pharmacologic inhibition of YAP signaling or ferroptosis significantly rescues POI. BNC1 is essential for maintaining mitochondrial function and oocyte lipid metabolism, while deficiency of BNC1 triggers iron-dependent POI. Perturbed expression of NF2 in BNC1 mutant mice results in dysregulation of oocyte redox homeostasis. Transcriptome data of Wang et al. showed that ferroptosis was involved in BNC1-induced POI. This uncover a pathologic mechanism of POI based on ferroptosis via YAP pathway.

Moreover, the clinical symptoms of polycystic ovary syndrome (PCOS) include oligomenorrhea, amenorrhea, hirsutism, increased risk of type 2 diabetes mellitus and insulin resistance (56, 57). Increased ROS production and decreased antioxidant capacity were observed in ovarian granulosa cells of PCOS patients; this is the cause of miscarriage and infertility in PCOS women (58, 59). PCOS-induced rats result in the activation of ferroptosis cascade and mitochondrial dysfunction in both uterus and placenta, which is associated with the triggering of GPX4/glutathione regulated lipid peroxidation and mitochondria-mediated ferroptosis (60). Both cross-sectional case-control studies (61), and meta-analysis (62) revealed high serum levels of ferritin, iron concentration, and hepcidin (an important regulator of iron homeostasis) in PCOS patients. As PCOS is associated with insulin resistance (63), disrupted iron metabolism may involve endocrine and metabolic disturbances rather than defects in hepcidin production. Therefore, it is not surprising that iron overload is associated with follicle dysplasia in PCOS patients. In sum, iron overload is tightly related to follicle development disorder characterized by ferroptosis, lipid peroxidation and oxidative stress.

## Hippo/YAP pathway regulates follicle development

Hippo pathway controls cell fate and organ growth through kinase complexes with evolutionarily conserved profiles (19). Kinase cascades are formed by mammalian sterile-20 like serine/threonine kinase 1/2 (MST 1/2), Salvador (SAV), and large tumor suppressor 1/2 (LATS 1/2). Downstream core effector YAP and transcriptional co-activator PDZ-binding motif (TAZ) are suppressed by the phosphorylation of these kinase complexes. Phosphorylated YAP/TAZ complex resulting in its cytoplasmic ubiquitin and degradation leads to nuclear transcription failure (64). In contrast, disrupted Hippo signaling urge unphosphorylated YAP/TAZ locates in the nucleus and induces expression of CCN family growth factors and baculoviral inhibitors of apoptosis repeat-containing (BIRC) apoptosis inhibitors by primarily binding to transcriptional enhanced associate domain (TEAD) transcription factors, to achieve the purpose of follicle development (20).

Evidence supports the key value of Hippo signaling pathway in follicle activation and growth. MST1/2, LATS1/2, and YAP/TAZ are expressed at different stages of follicle development in mouse and human ovaries (65–68). *In vitro* research reported that LATS1, during follicle development, can directly phosphorylate forkhead L2 (FOXL2), a vital regulator expressed in granulosa cells participating in follicle maturation, thereby controlling its transcriptional activity (69). Reduced primordial and activated follicles were observed in *Lats1* mutant (deletion) mouse ovaries, indicating its significance in maintaining ovarian reserve (70). YAP/TAZ signaling pathway is active *in vivo* (71), and supports proper follicle growth (72). Impaired follicle development and oocyte maturation were observed in *Foxl2*-driven *Yap*-deficient mice (73). Nagashima et al. observed abnormal follicle development (reduced preantral follicle number) in connective tissue growth factor (CTGF, a member of the CCN family) ovarian and uterine conditional knockout (cKO) mice (74). Additionally, the YAP gene is highly expressed in ovaries of PCOS women (75). This may be due to the increased YAP mRNA and protein levels caused by hypomethylation of the YAP promoter in PCOS patients (76). This is consistent with the hypothesis proposed by Dupont et al., that cell fate is induced by the rigid ECM where YAP/TAZ is active, and therefore YAP/TAZ function is required (77). In sum, the tangled Hippo web is an indispensable factor in follicle development obstruction.

## The upstream signal ECM governs follicle development via Hippo/YAP pathway

ECM is an extracellular network composed of macromolecules, which is related to the biomechanical support—the exchange of extracellular signals governing folliculogenesis and appropriate oocyte maturation (78). Disturbance of ECM homeostasis may lead to interference of biochemical pathways responsible for connecting cumulus-oocyte complex (COC), leading to impaired follicle development (79). Dynamic interactions exist between the ovarian biomechanics and microenvironment. Deposition and remodeling of matrix components are associated with early follicle activation (80). Primordial follicles are compressed and are in a state of intensive mechanical stress resulting from surrounding rigid ECM secreted by granulosa cells (15). The stiff physical environment—collagen-rich ovarian cortex is believed to be essential for maintaining follicle dormancy (64). Conversely, primordial follicles are activated on a soft matrix caused by ECM-degrading enzymes (15), which is consistent with the principle of ovarian fragmentation to improve rigid ovarian environments (20). A randomized controlled trial (RCT) assigned 34 women with poor ovarian response to receiving ovarian fragmentation or with no intervention. Although no significant difference was found in IVF outcomes, follicle activation was observed in patients undergoing ovarian fragmentation surgery (81). The Hippo pathway is extracellularly regulated by mechanical forces generated by ECM upstream signal (19), which is transduced by cytoplasmic actin (82). As a multifunctional protein, actin forms microfilaments to regulate follicle activation and development (65, 83–85). Once the globular actin (G-actin) polymerizes in stress fibers to form the filamentous actin (F-actin), the Hippo signaling pathway was disrupted (86). Cheng et al. successfully stimulated follicle growth in mice using jasplakinolide (JASP) or sphingosine-1-phosphate (S1P), which promoted actin polymerization and increased the F-actin/G-actin ratio, as well as subsequently activated YAP nuclear accumulation following the increase of CCN2 transcriptional level (83). Similarly, S1P addition to the culture medium reduced follicle atresia and improved the quality of primordial follicle (87–89). Hence, as an extracellular mechanical signal, ECM stiffness effectively regulates YAP/TAZ nuclear shuttling via dynamically intracellular actin dynamics, which controls the expression of CCN growth factors and BIRC apoptosis inhibitors in the nucleus, thus determining follicle fate. These findings pave the way for ECM protein levels to be a possible therapeutic target for follicle dysplasia.

## Iron-mediated ECM remodeling and follicle development

In the presence of excessive ECM, reduced matrix elasticity and rigid stiffness-induced mechanical stress are generated, thus interfering with the normal cellular biological behavior (90, 91). Positive evidence exhibits that within acceptable thresholds, iron regulates ECM production, including the synthesis of collagen (14, 92) and elastin (93). Higher intracellular iron levels may perturb the expression of genes encoding ECM components (94), suggesting that iron levels mediate matrix remodeling and degradation. Bunda et al. (93) demonstrated that elastin production was positively promoted in the range of 2-20μM iron concentration, yet reduced elastin output was observed when concentration goes out of scope. This is confirmed in the pathological mechanism of fibrosis. Several studies have reported the effect of iron metabolism on fibrosis progress. As a pathological phenomenon of chronic liver disease, liver fibrosis is characterized by excessive deposition of ECM with a typical observation of iron overload (95). Liver iron concentration over 60µmol/g can lead to abnormal function of hepatic stellate cell (HSC), and the threshold for transformation from fibrosis to cirrhosis is over 250µmol/g (96). Zhu et al. established a mouse model of liver fibrosis using carbon tetrachloride, suggesting a link between ferroptosis and liver fibrosis in HSC (97). Yuan et al. showed that triggering HSC ferroptosis alleviates liver damage and fibrosis since HSC is a major contributor to generating fibrosis (98). As a key signal mediating fibrosis, studies showed that iron increases the expression of TGF-β and collagen genes in HSC, thereby inducing fibrosis development (99, 100). Furthermore, during folliculogenesis, integrin mechanoreceptors on the plasma membrane of granulosa cells sense collagen concentration in cortical ECM and regulate the mechanoconduction cascade of Hippo signaling pathway according to the degree of rigidity (20, 85). Concentrations of other ECM components in follicular fluid, such as laminin and fibronectin, were positively correlated with oocyte competence in preovulation events (101). Oocytes synthesize bone morphogenetic protein 15 (BMP-15) and growth differentiation factor 9 (GDF9) belonging to the transforming growth factor β (TGFβ) superfamily, which seek for their respective receptors on granulosa cells to produce a soft visco-elastic ECM via SMAD2/3 or SMAD1/5/8 signalling, thus biomechanically paving the way for COC expansion (102, 103). Fibronectin levels in human follicle fluid are closely related to follicle size and oocyte maturation, indicating its significance in the process of folliculogenesis (104). Alahari et al. reported that under the condition of preeclampsia, iron regulates fibronectin assembly in primary mesenchymal stem cells, and abnormal ECM deposition blocks the migration of HTR-8/SVneo cells (105). In the course of mouse ovulation, the induction of granulosa cell luteinization and cumulus expansion depends on the fibronectin-integrin pathway (106). As a core component mediating the interaction between cells and ECM, the integrin family is crucial in delivering extracellular signal—ECM into cells (107). Cell detaching from ECM regards as a trigger of ferroptosis (108–110). α6β4 integrin has been reported to protect cancer cells from ferroptosis induced by erastin (a ferroptosis inducer) and ECM detachment, possibly due to inhibition of ACSL4 expression by α6β4 (108). Hence, iron overload produces abnormal ECM structures that lead to decreased cell proliferation, adhesion, and motility. The consequence of ECM overproduction is most likely a synergistic action launched by iron overload and TGF-β signal activation. However, whether iron overload is an independent factor contributing to increased ECM output, especially during follicle development, remains to be further investigated.

## The association of Hippo/YAP with TGF-β signaling mediates ECM remodeling and has potential implications for follicle development referring iron metabolism

Although lacking studies on follicle development models, correlations between iron metabolism and YAP have been reported in other tissues. YAP is a major downstream effector that mediates oxidative stress or ROS initiation, and phosphorylation of YAP induced by 10μM emodin suppressed ferroptosis and oxidative liver injury (111). YAP is a transcriptional stimulator of ferroptosis-activating genes ACSL4 and TFRC (112); it can directly bind to the TFRC promoter region and target TFRC expression to regulate iron levels (113). TFRC has been reported to be expressed in oocytes (114, 115) and is regulated by estrogen (116). Elevated TFRC mRNA expression was found in patients with endometriosis with reduced oocyte retrieval (117), suggesting adverse outcomes in follicles exposed to a potentially toxic iron-related environment. Therefore, we speculated that the activation of TFRC induced by active YAP might be one of the causes of follicles with iron overload.

Further, the regulatory effect of TAZ on NOXs has been demonstrated, yet the consequences are still controversial. In renal cell carcinoma, activated TAZ up-regulates NOX4 levels and subsequently increases intracellular lipid ROS levels inducing ferroptosis (118). Similarly, TAZ directly regulates the target gene angiopoietin-like 4 (ANGPTL4) and causes ferroptosis in ovarian cancer by activating NOX2 (119). However, a study on oxidative damage of uterine decidua illustrates that TAZ acts as an antioxidant role in restoring mitochondrial function by inhibiting NOX-stimulated ROS production to improve oxidative damage in stromal cells (120). NOXs appear to exert an effect on oocyte developmental stimulation. In a study on oocyte senescence, the fact that reduced NOX4 expression was observed in women more than 40 years old (121). Another study reported that FSH-induced oocyte maturation *in vitro* is dependent on NOX-mediated ROS activation (122). Therefore, we speculate that appropriate ROS level-mediated oocyte maturation is of great significance, yet excessive ROS undoubtedly accelerates adverse events induced by iron overload. However, evidence is still needed on how TAZ regulates NOXs in follicle development.

Moreover, TGF-β superfamily is involved in a series of biological events during folliculogenesis (123). Activated TGF-β receptors directly phosphorylate SMAD proteins launching nuclear gene transcription (124). SMAD2/3 is normally activated by TGF-β receptors (125). SMAD1/5/8, on the other hand, is induced by BMPs ligand, which mainly mediates hepcidin expression (126). SMAD4 forms a complex with phosphorylated SMAD2/3 or SMAD1/5/8, which translocates to the nucleus and binds to promoters triggering the transcription of numerous genes (124, 126). TGF-β is an effective component that enhances the production of ECM proteins and plays a key role in matrix remodeling (127). TGF-β/SMAD2/3 activation is a typical fibrosis pathway that mediates ECM deposition (13, 127). Further, crosstalk between TGF-β and Hippo signaling plays a synergistic role in transcriptional regulation (24). YAP and TAZ bind to SMAD proteins and are involved in the regulation of BMP or TGF-β signaling through different mechanisms (24). The two WW domains of YAP effectively bind to the PPxY motif in SMAD1 (128). In response to TGF-β signaling, TAZ and YAP are regulated by heteromeric SMAD2/3/4 complexes and determine their intracellular sublocalization (125). TGF-β and Hippo signals converge on transcriptional regulation of common target genes, for instance, CTGF, a component of surrounding ECM production that facilitates follicle assembly and maturation (129, 130).

TGF-β and Hippo pathway, therefore, are jointly aligned in the light of determining follicle fate through ECM remodelling, and may regard as key factors contributing to iron homeostasis.

## The hypothetical model

**Figure 1:**
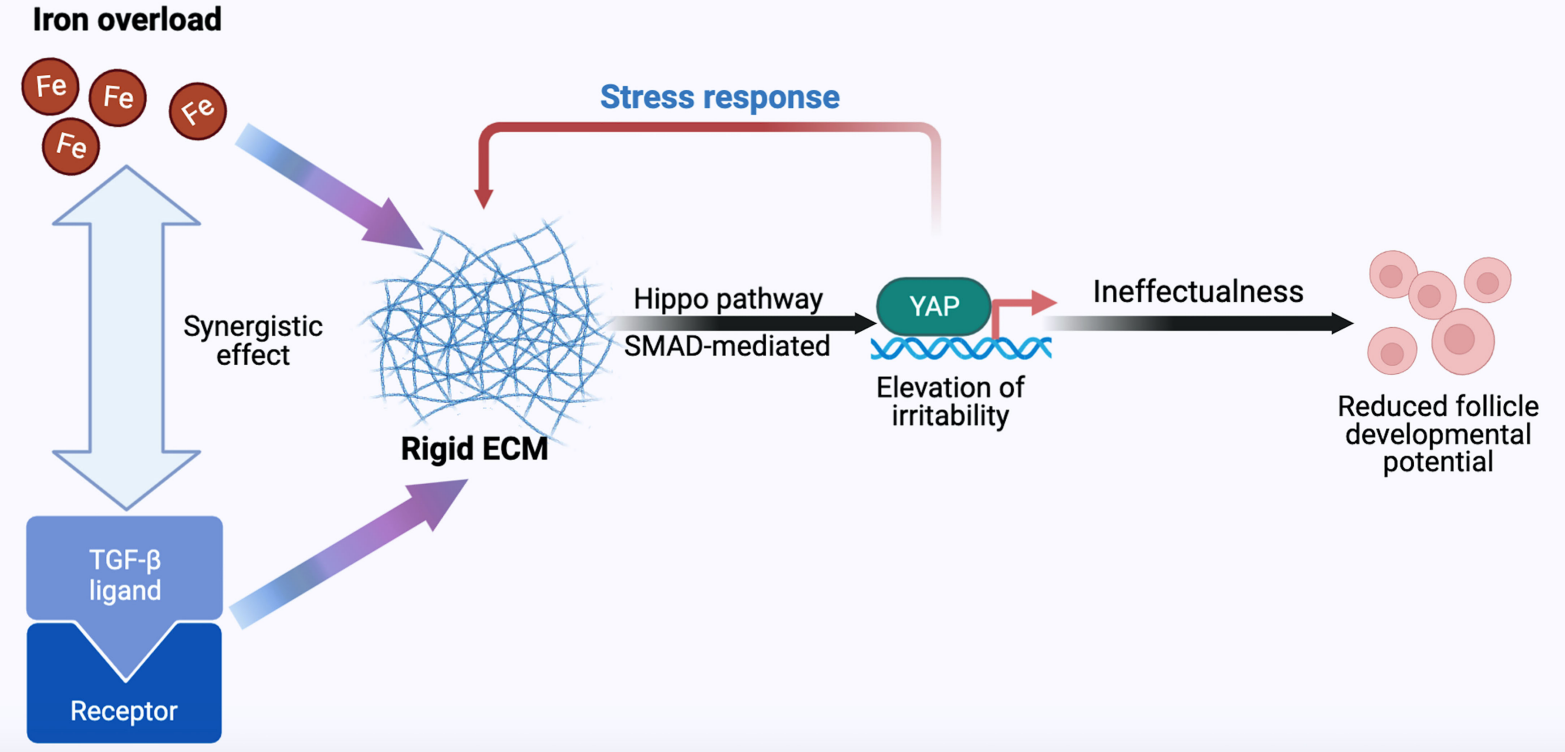
Hypothetical model of follicle developmental regulation. As the main effector, YAP responds to iron, ECM, Hippo, and TGF-β signals, thereby determining the follicle fate. Fe, iron; TGF-β, transforming growth factor-β; ECM, extracellular matrix; YAP, Yes-associated protein.

We emphasize that dynamic changes in terms of iron metabolism, ECM production, Hippo, and TGF-β/SMAD signaling pathways are involved in follicle development. The indisputable fact is that negative effort is made by excessive iron on follicle development. The activation of the TGF-β signal was shown to be caused by excess iron (100), and activated TGF-β up-regulates TFRC through the Hippo signaling pathway, enhancing intracellular accumulation of unstable iron and promoting fibrosis transition (131). Therefore, we proposed that overloaded iron and TGF-β signal have a synergistic effect through positive feedback to jointly stimulate the production of follicle-surrounding ECM, which is secreted by granulosa cells (23, 132). Increase in YAP/TAZ expression and its nuclear localization has been demonstrated and is regulated by TGF-β in patients with fibrosis (131, 133, 134). Through the binding of YAP/TAZ and SMADs, crosstalk occurs, promoting YAP/TAZ nuclear translocation under the activation of SMADs (125). ECM production is likely to be induced to increase, suggesting a possible scenario where matrix remodeling and mechanical stress occurr. The rigid ovarian cortex is wide open to compresse follicles; this is involved in the formation of actin stress fiber and integrin-mediated ECM-cell interactions. These changes increase mechanical forces and matrix stiffness on the periphery of follicles. Mechanical transduction also participates in nuclear dynamics in the regulation of YAP (135). The stress fibers generated on the stiff matrix deliver mechanical forces to the nucleus, followed by the flattened nuclear shape (15). Reduced mechanical limitations in nuclear pores appears to increase the YAP nuclear accumulation. By comparison, mechanical forces hardly transduced to the nucleus on the soft matrix, where nuclear YAP shuttling from cytoplasmic import via nucleus pore is equalized. The temporarily activated YAP/TAZ ultimately may not combat the rigid ovarian environment on account of positive cooperativity of TGF-β signal and iron overload, resulting in decreased follicle developmental potential (**Figure 1**).

**Figure 2:**
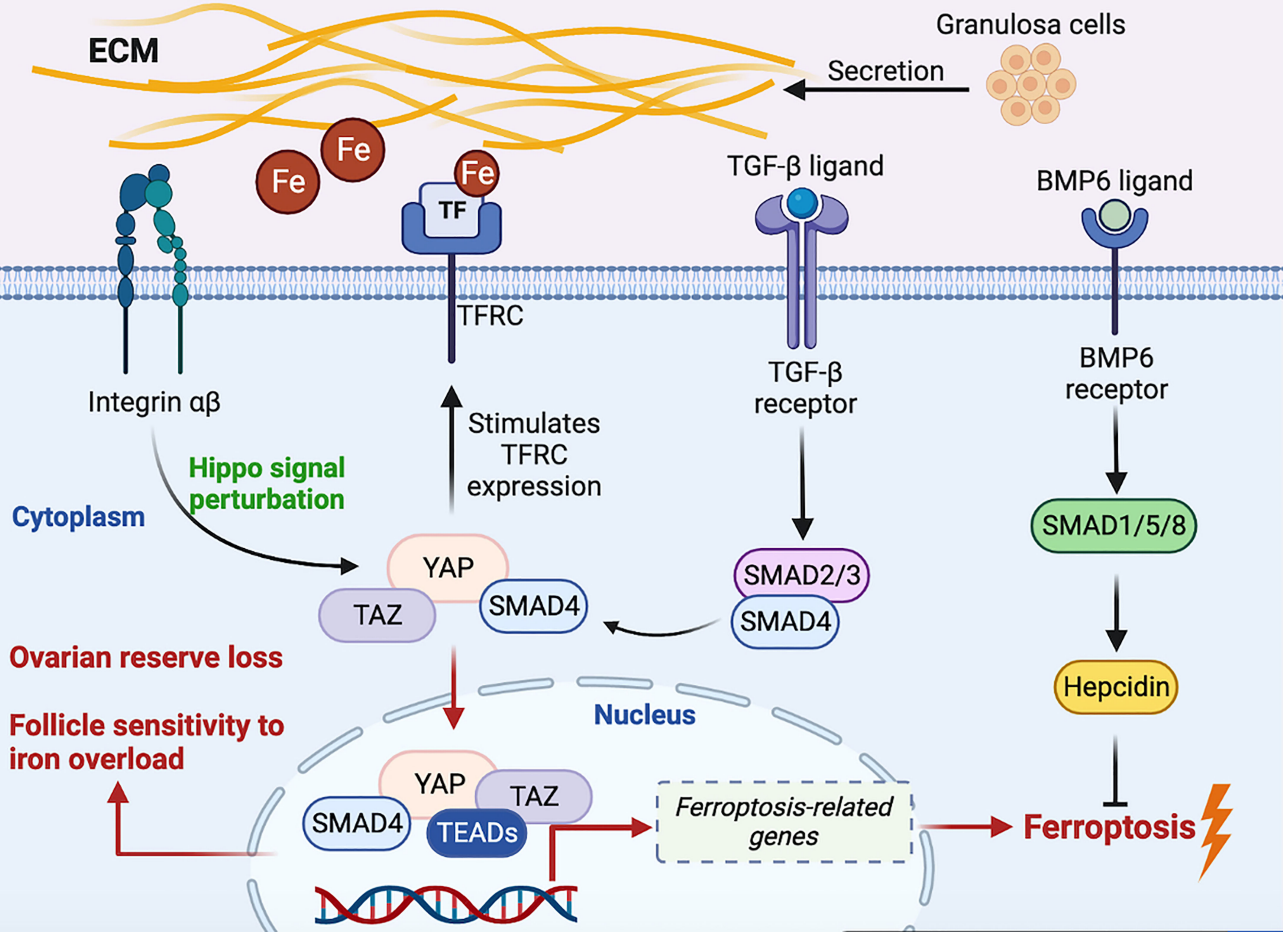
Hypothetical molecular signal model on regulatory iron metabolism of the intra- and extra-oocyte. Fe, iron; TGF-β, transforming growth factor-β; ECM, extracellular matrix; YAP, Yes-associated protein. TAZ, transcriptional co-activator PDZ-binding motif; TEADs, transcriptionally enhanced associate domains; BMP6, bone morphogenetic protein 6.

The Hippo/YAP pathway, TGF-β signal and iron metabolism are illustrated to regulate follicle growth (4, 5, 20, 23, 65), and TFRC is proved to be expressed in follicles and plays a vital role in follicle developmental trajectory (114, 116, 136). Therefore, from the perspective of intracellular environment, we hypothesize that a regulatory mechanism of iron metabolism is expected in follicles. YAP regulates iron metabolism by targeting TFRC (113), we proposed that TFRC mediate extracellular iron transport into the follicle. Increased cytoplasmic free iron concentration causes NOXs-induced Fenton chemistry, which stimulated the overproduction of intracellular ROS (137, 138). YAP/TAZ complex traveling into the nucleus, combine with TEADs transcription factor to promote the expression of ferroptosis-related genes such as TFRC and ACSL4 (131), thus being supposed to accelerate follicle lipid peroxidation and the progression of iron overload. In contrast, the BMP6-mediated SMAD1/5/8-SMAD4 signal enables excessive free iron to be stored through hepcidin (139, 140), thereby being expected to weaken the development of the Fenton reaction in follicles. Restriction of YAP/TAZ nuclear accumulation suppresses follicle development, we assume that follicles may build up resistant to iron overload-induced cytotoxicity. On the other side, Hippo signaling pathway perturbations and YAP/TAZ nuclear translocation/activation triggers follicle development; however, the susceptibility to iron excessive is supposed to be increased. Therefore, we theorize that by virtue of permissive YAP, follicle over-activation and the cytotoxicity of follicle affected by susceptibility to excess iron synergistically lead to the loss of ovarian reserve (**Figure 2**).

## Therapeutic implications and strategies

The drugs or compounds highlighted here are required for corresponding to the potential targets we have listed above if clinical progress needs to be achieved. In the iron metabolism, ferrostatin-1 acts as an effective component to resist ferroptosis (141, 142), and can lower TFRC levels (143). It has been shown to protect neural tissue (144–146), heart (147–149), liver (53), lung (150), colon (151) and angiotensin II-induced inflammation (145). To study the mechanism of ovarian granulosa cell injury in patients with PCOS, Shi et al. (152) confirmed that ferrostatin-1 has a protective effect on ovarian granulosa cells by regulating methylation mode through the homocysteine-induced KGN cell injury model. Similarly, liproxstatin-1, another ferroptosis inhibitor (141), has been reported to delay the enucleation of rat embryonic erythrocytes and hinder their maturation (143), possibly due to the high iron requirement of erythrocytes to maintain hemoglobin synthesis (153). In addition, iron chelating agents bind free iron to increase iron storage and thus lower intracellular iron levels. Yun et al. reported that the combined addition of GSH and iron-chelating agent can reduce ROS levels and the death of Chinese hamster ovary cells (154). The effect of hepcidin against ferroptosis is similar to that of ferrostatin-1, it can inhibit ferroptosis induced by acute respiratory distress syndrome (ARDS) via reducing iron uptake (155). Elevated serum hepcidin levels were observed in infertile women (156), as well as the trend of increased BNC1 deficiency in POI (22), suggesting a stress response to the regulation of iron metabolism. Therefore, how to fine-regulate endogenous hepcidin levels seem to be a vital target for the treatment of follicle development disorders caused by disrupted iron metabolism in POI patients. As the main ligand controlling the expression of hepcidin, evidence showed that long-term exogenous administration of BMP6 promoted the expression of endogenous hepcidin to improve the serum hepcidin deficiency and biochemical iron overload in *Hfe*-/- induced hemochromatosis mice (157). TMPRSS6, a type 2 transmembrane serine protease produced by the liver, negatively regulates the expression of hepcidin through BMP/SMAD pathway and participates in the regulation of iron homeostasis (158). Lipid nanoparticles containing TMPRSS6 siRNA increased the expression of hepcidin in the liver and alleviate iron overload in mice with hereditary hemochromatosis and β thalassemia (159).

Either controlling ECM overproduction or degrading the rigid ovarian cortex is endowed with clinical significance to solve follicle developmental obstruction. The current mainstream approach is to stimulate follicles through mechanical manipulation combined with *in vitro* activation therapy. POI ovarian cortex is surgically fragmented followed by autologous transplant, or the follicle development can be partially restored by *in vitro* culture in the presence of protein kinase B (AKT) stimulator before artificial transplant (65). These disrupted procedures, together with activation *in vitro*, promote actin polymerization and Hippo signal perturbation, followed by follicle growth (65, 81, 83). this is applied for rendering the successful pregnancy to infertile patients (160, 161). Given the presence of preoperative anxiety and fear of anesthesia in patients (162, 163), seeking for new compounds or drugs as an adjunct or even alternative to surgical therapies needs to be addressed. As mentioned above, treatment of mouse ovarian or human granulosa cells with agents that stimulate actin polymerization, such as JASP and/or S1P, increases nuclear YAP localization and downstream CCN growth factor expression, as well as activates follicle growth. In addition, YAP as a susceptibility gene may serve as a reliable target for screening PCOS (21). Since YAP/TAZ nuclear translocation can promote tumor development, dysregulation of YAP/TAZ signal transduction has been an effective drug target for inducing tumor cell apoptosis (164, 165). However, it remains to be seen whether direct YAP/TAZ regulators can be used in the reproductive field. Widely expressed YAP/TAZ requires specific drug-delivering carriers to gonads tissue following exerting its influence. Although YAP/TAZ nuclear translocation promotes follicle activation, excessive follicle activation due to high consumption of this complex may result in irreversible ovarian reserve failure, including POI disease. Therefore, how to delicately balance nucleocytoplasmic shuttling of YAP/TAZ is still an intractable issue on governing follicle development and dormancy. More evidence is needed on ameliorating the rigid ovarian microenvironment to optimize infertile therapy.

The disorder of iron metabolism in follicles is inseparable from the excessive production of oxidative stress products—cytoplasmic ROS, which can be weakened or reversed via being supplemented with antioxidants. Coenzyme Q10 (CoQ10) is an important lipid-soluble antioxidant in the human body (166). Doll et al. (167),reported that the inhibition of ferroptosis *via* ferroptosis suppressor protein 1 (FSP1) is mediated by CoQ10, which make up for GPX4 deficiency. FSP1 made NAD(P)H catalyze the regeneration of CoQ10. The FSP1-CoQ10-NAD(P)H pathway synergistically works with GPX4 and glutathione to inhibit ferroptosis. Administration of CoQ10 significantly increases the oocyte maturation rate in women aged 38-46 years, with reduced the oocyte aneuploidy rate and chromosome aneuploidy (168). Similarly, a randomized controlled study involving 186 subjects with reduced ovarian reserve demonstrated that CoQ10 supplementation during IVF cycles improved ovarian response, follicles count, and embryo quality (169). Resveratrol (RSV), another natural non-flavonoid polyphenol compound, can improve oocyte chromosome arrangement and spindle morphology, and have a positive effect on oocyte quality and quantity, as well as increase ovarian reserve to prolong ovarian lifespan (170). The recovery effect of RSV on ferroptosis has been widely established (171–174). RSV significantly restored the consumption of exogenous iron on follicle-stimulating hormone (FSH) and luteinizing hormone (LH), and decreased malondialdehyde (MDA, a product of membrane lipid peroxidation positively associated with ferroptosis) content (175). Additionally, it can effectively reduce oxidative stress and apoptosis of granulosa cells and oocytes in rats (176). In the same study, serum MDA was reduced in the resveratrol treatment group (176), indicating its significance in inhibiting iron overload in oocytes. Moreover, melatonin, as a free radical scavenger and broad-spectrum antioxidant produced by the pineal gland, serves an indispensable role in oocyte maturation, embryo development, and luteinization of granulosa cells (177). Similarly, role of melatonin in resisting iron overload has been extensively studied (178–181). Melatonin treatment significantly improves oocyte quality during IVF cycles, possibly by reducing ROS in oocytes and increasing GSH levels, as well as upregulating the expression of key genes being conducive to follicle developmental potentials, such as BMP-15, GDF-9, and GPX4, with down-regulating the expression of caspase-3 and other apoptotic genes (182).

In short, plenty of research on the mechanism of follicle development disorder remains in animal experiments. Iron overload and related molecular signals can be targeted therapeutically through different mechanisms (22) in POI. Although relevant evidence has yet to be investigated, multiple targets may be regulated via rational drug combination; this provides novel enlightenment for the subsequent drug development process.

## Summary and outlook

The present review highlights the significance of iron metabolism in follicle development and theorizes the underlying molecular mechanism that lead to follicle development disorders. Clues to the interaction between the Hippo pathway and iron overload sensitivity are integrated into follicle development events mediated by external environmental stimuli (ECM dynamics). Our hypothesis highlights that the combination of targeting TGF-β and iron overload may play a synergistic role in ECM overloading through the YAP/TAZ. In addition, we theorize a dynamic follicular iron homeostasis interacting with YAP, whose overactivation positively increases the risk of ovarian reserve loss and presumably enhances the follicle sensitivity to excess iron. However, the inferences are based on the existing evidence, and follow-up requires persuasive experimental confirmation and discussion. The mechanism of iron metabolic regulation and related signal transduction pathways applying to follicle development needs to be clarified. The corollary that hepcidin signals in response to follicle growth deserve further investigation. Blocking iron excess by various ferroptosis inhibitors or antioxidants may interfere with the progress of follicle dysplasia and may serve as an effective target for reducing adverse outcomes. We believed that YAP is a highly likely target for regulation of iron metabolism addressing ECM overproduction, which may therapeutically provide new insight into follicle developmental disorder. The role of iron in ECM deposition needs to be further determined; this may contribute to developing an iron-related diagnosis, prognosis, and treatment strategies in terms of aberrant follicle development.

## Author contributions

LX and YS contributed to manuscript writing and editing. SL and JD revised the manuscript for important intellectual content. All authors listed have made a substantial, direct, and intellectual contribution to the work and approved it for publication.
